# Survey of ticks and tick-borne pathogens in wild chimpanzee habitat in Western Uganda

**DOI:** 10.1186/s13071-022-05632-w

**Published:** 2023-01-22

**Authors:** Camille Lacroux, Sarah Bonnet, Emmanuelle Pouydebat, Marie Buysse, Nil Rahola, Sabine Rakotobe, John-Paul Okimat, Rachid Koual, Edward Asalu, Sabrina Krief, Olivier Duron

**Affiliations:** 1 UMR 7206 CNRS/MNHN/P7, Eco-anthropologie, Museum National d’Histoire Naturelle, Musée de l’Homme, 17 Place du Trocadéro, 75116 Paris, France; 2 Sebitoli Chimpanzee Project, Great Ape Conservation Project, Kibale National Park, Fort Portal, Uganda; 3 UMR 7179 CNRS/MNHN, Mécanismes Adaptatifs et Evolution, Museum National d’Histoire Naturelle, 57 Rue Cuvier, 75231 Paris, France; 4 La Phocéenne de Cosmétique, ZA Les Roquassiers, 174 Rue de la Forge, 13300 Salon-de-Provence, France; 5 UMR 2000, Ecology and Emergence of Arthropod-Borne Pathogens, Institut Pasteur/CNRS/Université Paris-Cité, 75015 Paris, France; 6 Animal Health Department, INRAE, 37380 Nouzilly, France; 7 UMR 5290 MIVEGEC (Maladies Infectieuses et Vecteurs : Ecologie, Génétique, Evolution et Contrôle), CNRS/IRD/Université de Montpellier, 911 Avenue Agropolis, 34394 Montpellier, France; 8 MEEDiN (Montpellier Ecology and Evolution of Disease Network), Montpellier, France; 9 UMR BIPAR ANSES-INRAE-EnvA, Laboratoire Santé Animale, 94701 Maisons-Alfort, France; 10 Uganda Wildlife Authority, Plot 7 Kira Road, Kamwokya, Kampala City, Uganda

**Keywords:** *Amblyomma*, Piroplasmids, *Borrelia*, *Cryptoplasma*, *Ehrlichia*, *Rickettsia*, Apes, Ticks, Vector-borne pathogens, Kibale

## Abstract

**Background:**

Ticks and tick-borne pathogens significantly impact both human and animal health and therefore are of major concern to the scientific community. Knowledge of tick-borne pathogens is crucial for prescription of mitigation measures. In Africa, much research on ticks has focused on domestic animals. Little is known about ticks and their pathogens in wild habitats and wild animals like the endangered chimpanzee, our closest relative.

**Methods:**

In this study, we collected ticks in the forested habitat of a community of 100 chimpanzees living in Kibale National Park, Western Uganda, and assessed how their presence and abundance are influenced by environmental factors. We used non-invasive methods of flagging the vegetation and visual search of ticks both on human team members and in chimpanzee nests. We identified adult and nymph ticks through morphological features. Molecular techniques were used to detect and identify tick-borne piroplasmids and bacterial pathogens.

**Results:**

A total of 470 ticks were collected, which led to the identification of seven tick species: *Haemaphysalis parmata* (68.77%), *Amblyomma tholloni* (20.70%), *Ixodes rasus* sensu lato (7.37%), *Rhipicephalus dux* (1.40%), *Haemaphysalis punctaleachi* (0.70%), *Ixodes muniensis* (0.70%) and *Amblyomma paulopunctatum* (0.35%). The presence of ticks, irrespective of species, was influenced by temperature and type of vegetation but not by relative humidity. Molecular detection revealed the presence of at least six genera of tick-borne pathogens (*Babesia*, *Theileria*, *Borrelia*, *Cryptoplasma*, *Ehrlichia* and *Rickettsia*). The Afrotopical tick *Amblyomma tholloni* found in one chimpanzee nest was infected by *Rickettsia* sp.

**Conclusions:**

In conclusion, this study presented ticks and tick-borne pathogens in a Ugandan wildlife habitat whose potential effects on animal health remain to be elucidated.

**Graphical Abstract:**

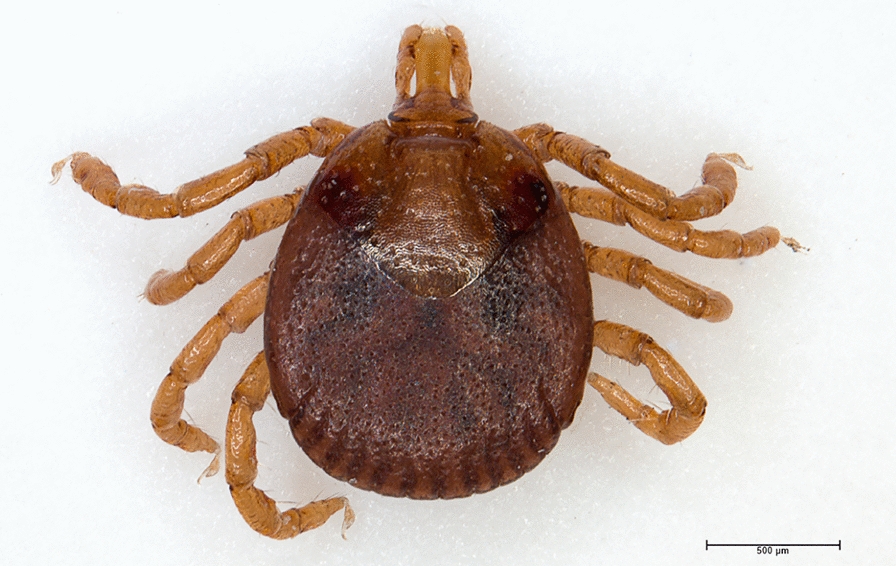

**Supplementary Information:**

The online version contains supplementary material available at 10.1186/s13071-022-05632-w.

## Background

In the tropical zone, most efforts are being made to understand the impacts of ticks and tick-borne pathogens on the health of humans [1–4] and domestic animals because of the associated economic losses [5–12]. In Africa, a great diversity of ticks is present [13], and some species are potential carriers of tick-borne pathogens of major importance. For instance, some *Amblyomma* species, a vector of *Rickettsia africae*, and *Rhipicephalus appendiculatus*, a vector of *Theileria parva*, cause African bite fever and East Coast fever, respectively, a huge hurdle to the development of the livestock industry [1, 13–15]. In parallel, ticks are known to feed on a large variety of vertebrate hosts including mammals, birds, reptiles and amphibians, but only a few studies have been performed on wild animals [16–20] since the first inventories [21–27].

The chimpanzee (*Pan troglodytes*) population has been estimated at around 345,000 individuals belonging to four sub-species across 21 countries [28]. According to the IUCN red list [29], our closest relative is an endangered species threatened with extinction in the near future, so understanding the factors affecting its health demands urgent attention. With the exception of three studies that reported tick infestation in the nostrils of chimpanzees and primatologists working in the Ugandan forest [30–32], little is known about the risks ticks may pose to chimpanzee health, also regarding their implication for human beings living or working in close proximity to ape habitats.

Chimpanzees, like other non-human primates, have protective methods to remove their ectoparasites like ticks. This behavior is called “grooming,” and some chimpanzees invest as much as a fifth of their time grooming [33]. A chimpanzee can practice self-grooming or allo-grooming. Mutual allo-grooming is part of affiliative relationships to reduce tension and aggression between individuals [34, 35]. A more direct aspect of allo-grooming is the altruistic hygienic function allowing access to body parts not accessible to self-grooming [36, 37]. The presence of hematophagous ectoparasites such as ticks can affect the body condition and fitness of individuals and thus the population health [38, 39]. In addition, these ectoparasites can be vectors of pathogens that may also impact chimpanzee health, but no studies have yet been done on this topic in wild conditions to our knowledge.

Host-seeking ticks position themselves on vegetation to detect hosts with their Haller’s organ by their shadow, body heat, odor and vibrations caused by their movement [40]. Searching for ticks in vegetation is therefore a good way to catch questing ticks in wild habitat, using the least invasive method possible. Every evening, like all great apes, weaned chimpanzees build a ‘nest’ by bending branches of a tree in which they will spend the night [41]. Exploring the nests in the morning just after the chimpanzees have left them provides a complementary opportunity to collect ticks to which the chimpanzees are exposed: non-questing ticks present in the vegetation or ticks from the fur of chimpanzees that have not yet attached or were removed by self-grooming.

A variety of biotic and abiotic factors are known to affect the development and survival rates of ticks. Indeed, their abundances are known to vary over time, both seasonally and annually, as well as spatially between habitats and ecological zones, because of the interactions of numerous factors, such as host diversity, changing vegetation structure and climate [42–45]. In addition, their host-seeking activity appears to be closely related to daily air temperature and relative humidity [46].

In this study, we assessed the presence of ticks and tick-borne pathogens in the habitat of wild chimpanzees in western Uganda where domestic animals are banned. First, we collected ticks using non-invasive methods according to chimpanzee behavior guidelines over 6 months and identified them. We determined how the number of collected ticks is influenced by season, vegetation type and daily temperature and humidity. Second, we used molecular techniques to detect tick-borne piroplasmids and bacterial pathogens and compared them with known pathogens using phylogenetic analyses for their identification.

## Methods

### Study site

Our study took place in the Sebitoli area located at the extreme northern part of the Kibale National Park in Western Uganda (795 km^2^, 0°13′-0°41′N and 30°19′-30°32′E1; [47]). This park is a mid-altitude forest with high plant and animal diversity [48]. The weather of this equatorial area comprises two rainy (from March to May and September to November) and two dry seasons (from December to February and June to August) [49]. The Sebitoli forested area is surrounded by many agricultural parcels such as small farms with some domestic animals (chickens, goats, a few cows, dogs and cats), food crops, and tea and eucalyptus plantations. The Sebitoli territory is composed of 70% secondary forest, 14% mature forest, 14% terrestrial herbaceous vegetation and 1% patchy shrub/wetland vegetation [50]. Since 2009, the Sebitoli Chimpanzee Project (SCP) team has been monitoring a chimpanzee (*Pan troglodytes schweinfurthii*) community of about 100 individuals daily, with 66 identified. A tarmac road cuts the 25 km^2^ community home range [50–52].

### Sampling design and collection of ticks

**Fig. 1:**
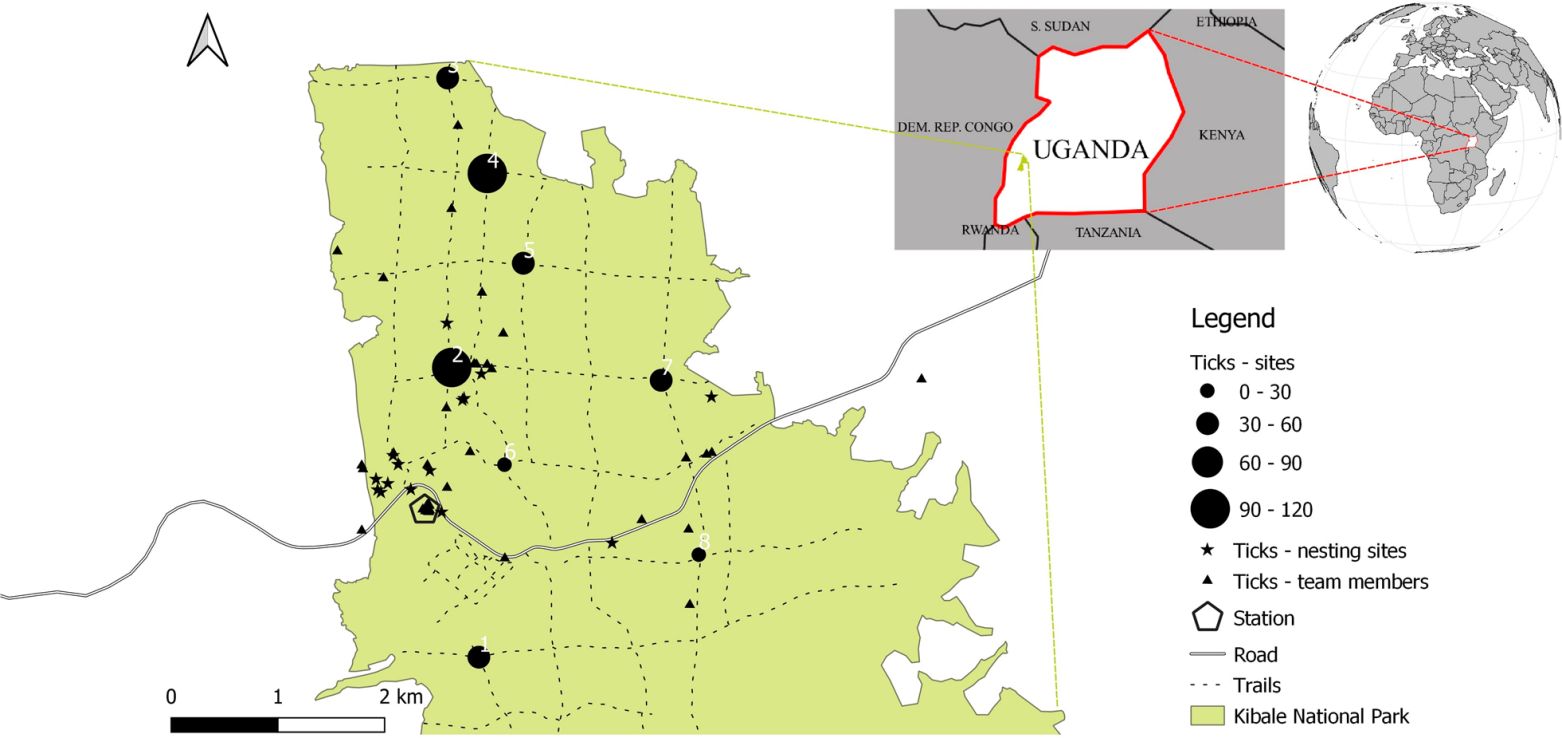
Tick collections across the Sebitoli chimpanzee habitat with the total number of ticks collected weekly in the eight studied sites, ticks collected at nesting sites and on team members

Four methods were used:

1. Flagging method at eight ‘vegetation sites’: a 50 cm × 50 cm white cotton flag was swept from side to side parallel to the ground over vegetation or ground litter in front of the collector in 2 m × 1 m quadrats. In each vegetation site, four quadrats by cardinal points at 3 m and four quadrats at 10 m from a randomly selected central point were analyzed. The eight vegetation sites were distributed in the chimpanzee habitat and represent the different types of vegetation present in the Sebitoli area (mature forest, secondary forest, terrestrial herbaceous vegetation and trail) (Fig. 1). Each quadrat of each vegetation site was systematically visited during the day once a week between 26 September 2019 and 21 March 2020 (i.e. 47 days of collection). To ensure consistency, two to three of the same collectors were assigned to this task and spent 2 min per sampling site.
2. Chimpanzee nest visual search: between 9 September 2019 and 21 November 2019, a total of 18 chimpanzee nests were searched for the presence of ticks. Only nests < 15 m high were climbed for security reasons. They were investigated by visual search to look for ticks present in the dense foliage within 3 h after the chimpanzee departure.
3. Flagging method at the nesting sites: the same flagging method was applied at nesting sites around the nesting tree in the morning after a chimpanzee had slept in it (Fig. 1). The central point was the base of the trunk of the nesting tree.
4. Fixed and non-fixed ticks were also taken from the body, clothing or bags of the research team members during the study between 9 September 2019 and 21 November 2019 (Fig. 1).

The temperature and the relative humidity of the collection day were obtained from the POWER Project (https://power.larc.nasa.gov/data-access-viewer/ on 2022/06/15).

### Tick identification

As soon as collected, ticks were preserved in a 70% ethanol solution. A taxonomic identification was done following the identification keys for Ugandan ticks written by Matthysse and Colbo [53] and Walker and colleagues [12] with some clarifications for the genus *Haemaphysalis* [54, 55]. Representative individuals were photographed by image stacking with a Leica Z16 APO-A macroscope as pictures of these specimens are lacking in the literature.

### Detection of tick-borne pathogens

Ticks used for further investigations were collected between 26 September 2019 and 16 January 2020. Ticks at adult and nymphal stages were processed individually; larvae were excluded.

To avoid external cuticular microbial contaminants, ticks were processed with commercial bleach diluted at 1% for 30 s and then rinsed for 1 min in three successive baths of DNA-free water following a published protocol [56]. For each tick specimen, total DNA was extracted from whole crushed body using a genomic DNA extraction kit according to the manufacturer’s instructions (DNeasy Blood and Tissue extraction kit, Qiagen, Hilden, Germany). Each individual DNA extract was then tested for the presence of piroplasmid and bacterial pathogens using distinct protocols.

To detect tick-borne piroplasmids, each individual DNA extract was tested by semi-nested polymerase chain reaction (PCR) by amplifying a 437–479-bp fragment of the 18S rRNA gene using piroplasmid-specific primers (listed in Additional file 1: Table S1). Positive (DNA of an *Amblyomma cajennense* tick infected by *Babesia*) and negative (sterile water) controls were included in each PCR assay. All positive PCR products were purified and sequenced in both directions, and then chromatograms were manually cleaned with CHROMAS LITE (http://www.technelysium.com.au/chromas_lite.html) to ensure that the record represented a true positive.

To detect tick-borne bacterial pathogens, we conducted a bacterial metabarcoding approach to screen all specimens efficiently and then to identify individuals carrying bacterial genera of medical and veterinary interest for further analyses. To this aim, a 251-bp portion of the V4 variable region of the bacterial 16S rRNA gene was then amplified individually for each individual DNA extract using a Multiplex PCR Kit (Qiagen, Hilden, Germany), as described by [56]. Amplified bacterial 16S rDNA products were individually tagged with a unique 35-base barcode using the Nextera Index Kit (Illumina, San Diego, CA, USA), purified and sequenced on an Illumina MiSeq platform (GenSeq, Montpellier University). All bioinformatic analyses were conducted using the pipeline FROGS (https://github.com/geraldinepascal/FROGS) mainly as follows by [56], considering that the clustering was here performed with an aggregation distance value of 3 and the sequences’ clusters were clustered together with a minimum identity value of 96% to define OTUs. To eliminate the possibility of contamination, we included ten DNA extraction controls and three amplification control representatives of the different pins, sterile water, buffers and kits used. To treat contaminants’ OTUs, if the count of an OTU in the sum of controls was > 50% of its count in the sum of samples, the OTU was removed. We obtained an average number of 19,053 bacterial 16S rDNA reads per tick specimen. According to the obtained abundance file and OTU assignation, a random subset of DNA templates with reads of bacteria of potential interest was further used for additional molecular typing and refined bacterial identification. These tick-borne bacteria, including *Rickettsia* sp., *Borrelia* sp., *Ehrlichia* sp. and *Cryptoplasma* sp., were each genotyped using specific semi-nested PCR assays (Additional file 1: Table S1). To prevent possible contamination, different parts of this process were physically separated from one another in entirely separate rooms.

Sequence alignments were performed using CLUSTALW [57], implemented in the MEGA7 software [58]. The GBLOCKS program with default parameters was used to remove poorly aligned positions and to obtain unambiguous sequence alignments [59]. The evolutionary models most closely fitting the sequence data were determined using Akaike information criterion with MEGA7 [58]. Phylogenetic analyses were based on maximum likelihood (ML) approach. A ML heuristic search, using a starting tree obtained by neighbor joining, was conducted, and clade robustness was further assessed by bootstrap analysis using 1000 replicates in MEGA7 [58].

All novel nucleotide sequences were deposited in the GenBank nucleotide database (accession numbers OQ092409-OQ092416, OQ092427-OQ092429, OQ096007-OQ096015).

## Data analysis

The numbers of ticks, tick species and tick pathogens were determined in each sampling site. To compare the mean abundance of ticks or their pathogens between seasons, a t-test was performed. Differences between multiple groups like vegetation types were examined using a one-way analysis of variance.

We fitted generalized linear models with a Poisson structure to investigate whether the temperature and relative humidity of the day and the vegetation type influenced the number of ticks [60, 61]. Before fitting the model, we tested for multicollinearity between variables. All continuous variables were scaled with a mean of 0 and standard deviation of 1 (i.e. z-transformation). We confirmed that there were no major internal correlation problems based on the variance inflation factor (VIF) (maximum VIF < 1.31) using the “vif” function from the “car” package [62]. We assessed the joint effect of the variables by comparing the *full* model, including the tested predictors as well as the control variables, to the null model, using a likelihood-ratio test (R-function ANOVA set to “Chisq” [60]). When this comparison was significant, we tested the singular effect of each variable by comparing the *full* model deviance and the deviance of a *reduced* model [63] excluding the variable of interest one by one, using the “drop1” function [64]. To account for the multiple testing effect, we kept the false discovery rate at the nominal value of 0.05 [65]. The samples included a total of 1551 points. We visually checked the models’ assumption (e.g. homogeneous distribution of residuals vs fitted values), and assessed the models' stability using various parameters such as leverage values (0.01), maximum Cook’s distance (0.30), maximum Dffits (0.79) and maximum DFBetas (0.73). It indicated no obviously influential cases [66]. Furthermore, overdispersion of the points was not an issue in our model (2.71).

All statistical analyses were performed in R software, version 3.6.0 [67].

## Results

### Occurrence of tick species

A total of 470 ticks (38 adults, 247 nymphs and 185 larvae) were collected with the three collection methods. The larvae were not identified and not considered further. For the adults and nymphs, seven species in four genera were recorded: *Haemaphysalis parmata* (68.77%), *Amblyomma tholloni* (20.70%), *Ixodes rasus* s.l. (7.37%), *Rhipicephalus dux* (1.40%), *Haemaphysalis punctaleachi* (0.70%), *Ixodes muniensis* (0.70%) and *Amblyomma paulopunctatum* (0.35%) (Table 1). Pictures of individuals from each species are available in Additional file 2: Figure. S1.

**Table 1:** Number of ticks identified according to their life stage, their sex and the site of collection

Species	Life stage and sex				Collection sites		
	Adult male	Adult female	Nym-ph	Lar-va	Vege-tation sites	Nesting sites/inside nests	Team membe-rs
*Amblyomma paulopunctatum* Neumann, 1899	1	0	0		0	0/0	1
*Amblyomma tholloni* Neumann, 1899	0	2	57		27	2/1	29
*Haemaphysalis parmata* Neumann, 1905	9	17	170		189	2/0	5
*Haemaphysalis punctaleachi* Camicas, Hoogstraal & El Kammah, 1973	1	1	0		1	0/0	1
*Ixodes muniensis* Arthur & Burrow, 1957	2	0	0		1	0/0	1
*Ixodes rasus* sensu lato (s.l.) Neumann, 1899	0	1	20		18	1/0	2
*Rhipicephalus dux* Dönitz, 1910	2	2	0		1	0/0	3
Unidentified				185	159	21/0	5
TOTAL	15	23	247	185	396	26/1	47

Ticks of different species, life stages and sexes were collected from several collection sites. One individual of *A. tholloni* was collected directly inside the fresh nest of a chimpanzee. At chimpanzee nesting sites, three species were found: *I. rasus* s.l, *A. tholloni* and *H. parmata.* At the eight vegetation sites across the Sebitoli area, all species were found except *A. paulopunctatum*. From the team members, all seven species were found (Table 1). Most of the ticks collected (84.26%) were obtain using the flagging method at vegetation sites, while only 5.53% were collected using the flagging method at nesting sites and 0.21% collected inside nests; 10.00% were collected on the bodies or equipment of team members (Table 1).

**Fig. 2:**
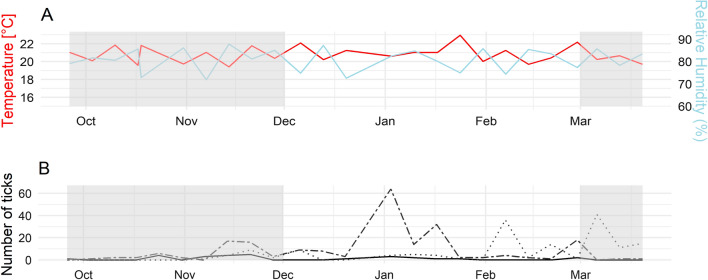
Weekly representation of meteorological factors (**A**) and tick stage abundance (**B**) in sites across the Sebitoli Chimpanzee Project inside the Kibale National Park, Uganda. Gray area represents the rainy season. **B** The plain line represents adults, the dashed line nymphs and the dotted lines larvae

During 3 months of weekly tick collections, we found temporal differences in tick numbers. We found more ticks during the dry season than during the rainy season (t-test: *t *= 3.9088, df = 1300.4, *p*-value < 0.0001). At the species level, only the abundance of *Haemaphysalis* spp*.* varied with season (t-test: *t* = 5.125, df = 1048.5, *p*-value < 0.0001). The most predominant tick life stage was nymphs around January, while larvae were found in numbers in February and March (Fig. 2).

### Tick-borne pathogen identification

Among the piroplasmid tick-borne pathogens, two protozoan genera were detected with semi-nested PCR: *Babesia* sp. and *Theileria* sp. Four additional bacterial genera of human and veterinary medicine interest were detected via high-throughput 16S rDNA sequencing: *Borrelia* sp., *Cryptoplasma* sp., *Ehrlichia* sp. and *Rickettsia* sp.

Of 220 adults and nymphs, 30.91% were positive for *Rickettsia* sp. (*A. tholloni*, *n* = 43; *I. rasus* s.l., *n* = 17; *H. parmata*, *n* = 5; *A. paulopunctatum*, *n *= 1; *I. muniensis*, *n *= 1; *Rh. dux*, n = 1) and 6.81% for *Cryptoplasma* sp. (*A. tholloni*, n = 12; *H. parmata*, n = 3); 2.73% were positive for *Theileria* sp. (*A. tholloni*, *n* = 6) and 1.82% for *Borrelia* sp. (*H. parmata*, n = 4); 0.91% were positive for *Babesia* sp. (*H. parmata*, *n* = 1; *I. muniensis*, *n* = 1) and 0.45% for *Ehrlichia* sp. (*H. punctaleachi*, *n* = 1).

**Fig. 3:**
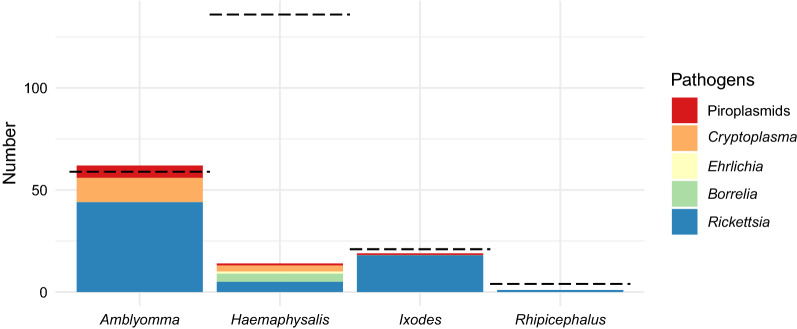
Tick-borne pathogens found in ticks according to their genus. The dashed line represents the total number of individuals collected. One tick can carry multiple pathogens

Of 59 *Amblyomma* spp., 74.58% were positive to *Rickettsia* sp., 21.42% to *Cryptoplasma* sp. and 10.17% to *Theileria* sp. Of 136 *Haemaphysalis* spp., 3.68% were positive to *Rickettsia* sp., 2.21% to *Cryptoplasma* sp., 0.74% to *Ehrlichia* sp. and 0.69% to *Babesia* sp. Of 21 *Ixodes* spp., 85.71% were positive to *Rickettsia* sp. and 4.76% to *Babesia* sp. Finally, of four *Rhicephalus* spp., 25.00% were positive to *Rickettsia* sp. and none to piroplasmids (Fig. 3).

Of 171 adults and nymphs collected at vegetation sites, 30.41% were positive for at least one pathogen. Of five adults and nymphs collected at the nesting site, 40.00% were simultaneously positive to *Rickettsia* sp. and *Cryptoplasma* sp*.* The nymph of *A. tholloni* collected inside the chimpanzee nest was infected with *Rickettsia* sp. Of 42 adults and nymphs collected from team members, 64.29% were positive for at least one pathogen.

### Environmental determinants of tick and pathogens abundance

**Fig. 4:**
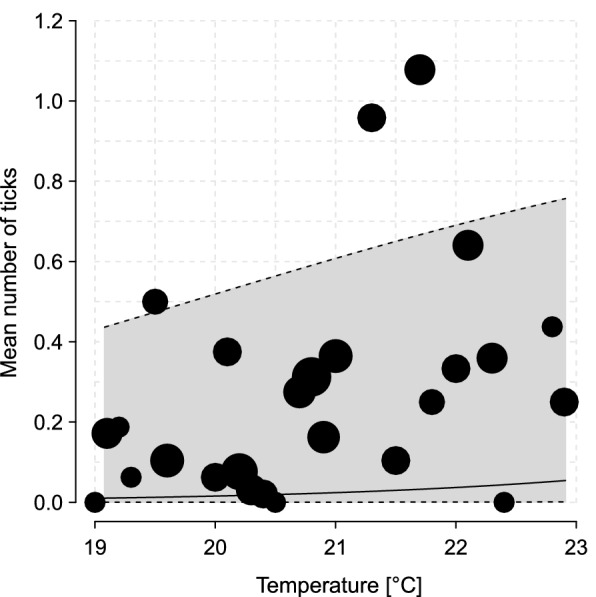
Influence of temperature (°C) on the mean number of ticks in sites across the Sebitoli chimpanzee habitat. The black dots represent the average of the raw data. The solid black line represents the model response, which depicts the influence of temperature while averaging the effects of all other variables. The gray polygon indicates the 95% confidence interval; borders are represented by the black dashed lines

Number of ticks was significantly affected by daytime temperature and by vegetation type but not by the relative humidity (Table 2). Tick abundance increased with temperature (Fig. 4).

**Table 2:** Results of the GLM on the number of all ticks collected

Term	Estimate	Standard deviation (SD)	Degree of freedom (df)	Statistics value (χ^2^)	*p*-value
Ticks model (*n* = 1551)					
Intercept	− 24.00	4.43			
Temperature.z	4.16	0.59	1	49.64	< 0.0001 ***
Humidity.z	0.31	0.24	1	1.72	0.1895
Type.vegetation	1.13	0.27	3	31.02	< 0.0001 ***

“.*z*” predictors were *z*-transformed to a mean of zero and a standard deviation of one. Ticks model: response is the number of ticks collected in a sampling site. Temperature: mean temperature measured in  °C from the day of collection. Humidity: the percentage of relative humidity measured in % from the day of collection. TypeVegetation: type of vegetation characterizing the site’s square (primary forest, secondary forest, trail or herbaceous vegetation)

A gradient in ticks according to the vegetation was found, with a mean number of ticks more important in the trail system (0.48), then the herbaceous vegetation outside the trail system (0.33) and the secondary forest (0.30) and finally less in the primary forest (0.11) (ANOVA, F-value = 2.969, *p*-value = 0.0309). There was a significant difference in the number of pathogen genera between tick life stages (t-test: *t* =  − 2.423, df = 62.8, *p*-value = 0.0183). Nymphs had more pathogens than adults.

### Tick-borne pathogen phylogenetic analyses

**Fig. 5:**
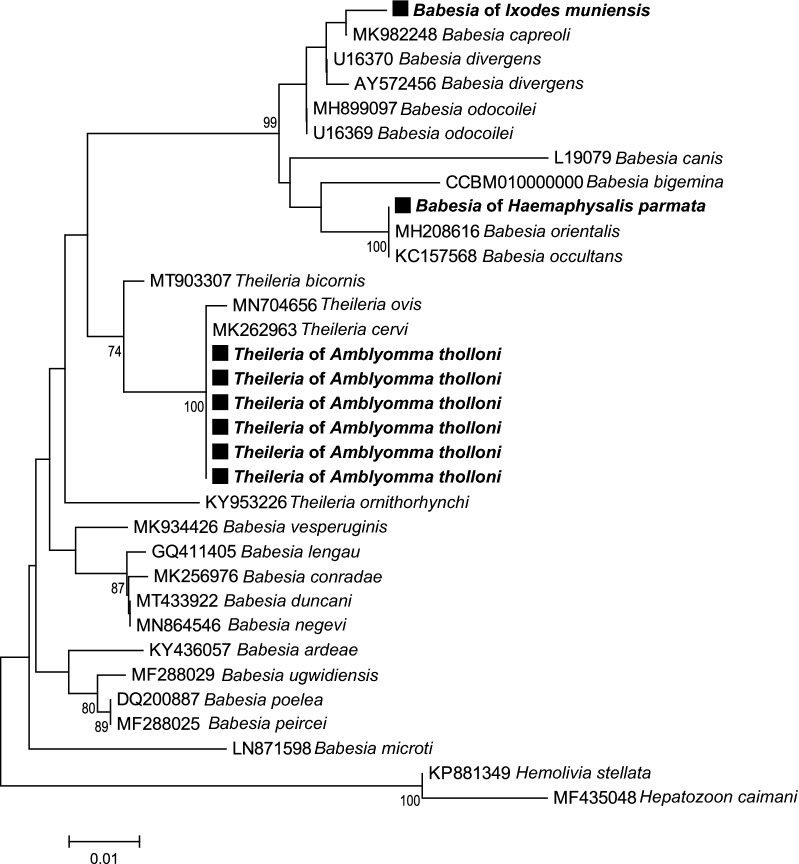
Piroplasmid phylogenetic tree constructed using maximum-likelihood (ML) estimations based on 18S rDNA nucleotide sequences (350 unambiguously aligned bp; best-fit approximation for the evolutionary model: T92 + G), including representative *Babesia*, *Theileria*, *Hemolivia* and *Hepatozoon* species (their GenBank accession numbers are indicated on the tree). Branch numbers indicate percentage bootstrap support (1000 replicates). Only bootstrap supports > 70% are shown. The scale bar is in units of substitution/site. Black squares indicate piroplasmid sequences obtained in this study

**Fig. 6:**
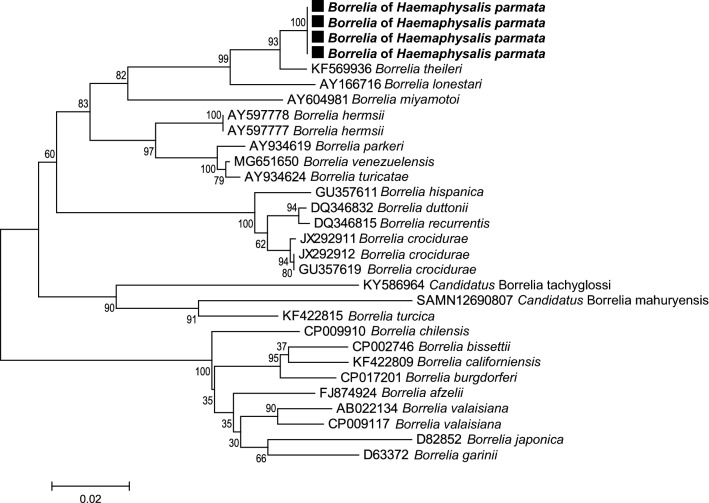
*Borrelia* phylogenetic tree constructed using maximum-likelihood (ML) estimations based on *flaB* nucleotide sequences (438 unambiguously aligned bp; best-fit approximation for the evolutionary model: T92 + G), including representative *Borrelia* species (their GenBank accession numbers are indicated on the tree). Branch numbers indicate percentage bootstrap support (1000 replicates). Only bootstrap supports > 70% are shown. The scale bar is in units of substitution/site. Black squares indicate Borrelia sequences obtained in this study

**Fig. 7:**
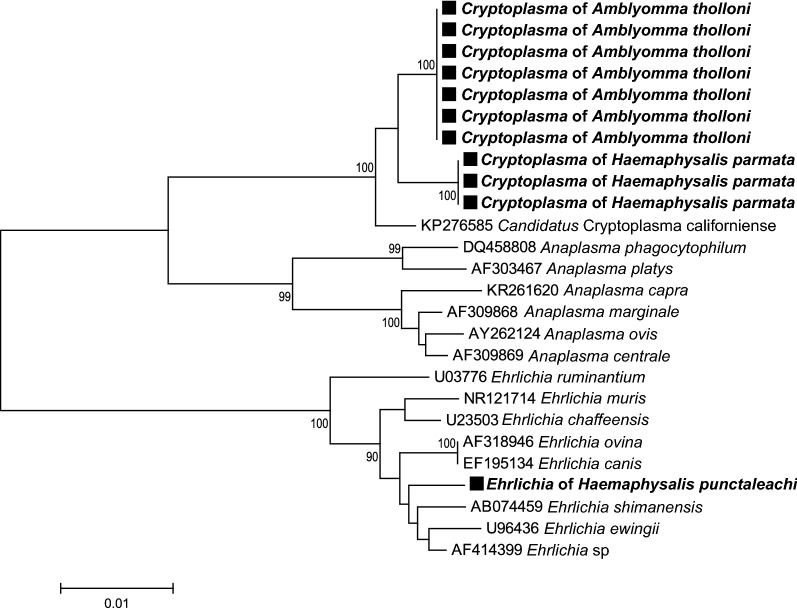
*Cryptoplasma* and *Ehrlichia* phylogenetic tree constructed using maximum-likelihood (ML) estimations based on 16S rDNA nucleotide sequences (1148 unambiguously aligned bp; best-fit approximation for the evolutionary model: K2 + G), including representative *Cryptoplasma*, *Ehrlichia* and *Anaplasma* species (their GenBank accession numbers are indicated on the tree). Branch numbers indicate percentage bootstrap support (1000 replicates). Only bootstrap supports > 70% are shown. The scale bar is in units of substitution/site. Black squares indicate Cryptoplasma and Ehrlichia sequences obtained in this study

**Fig. 8:**
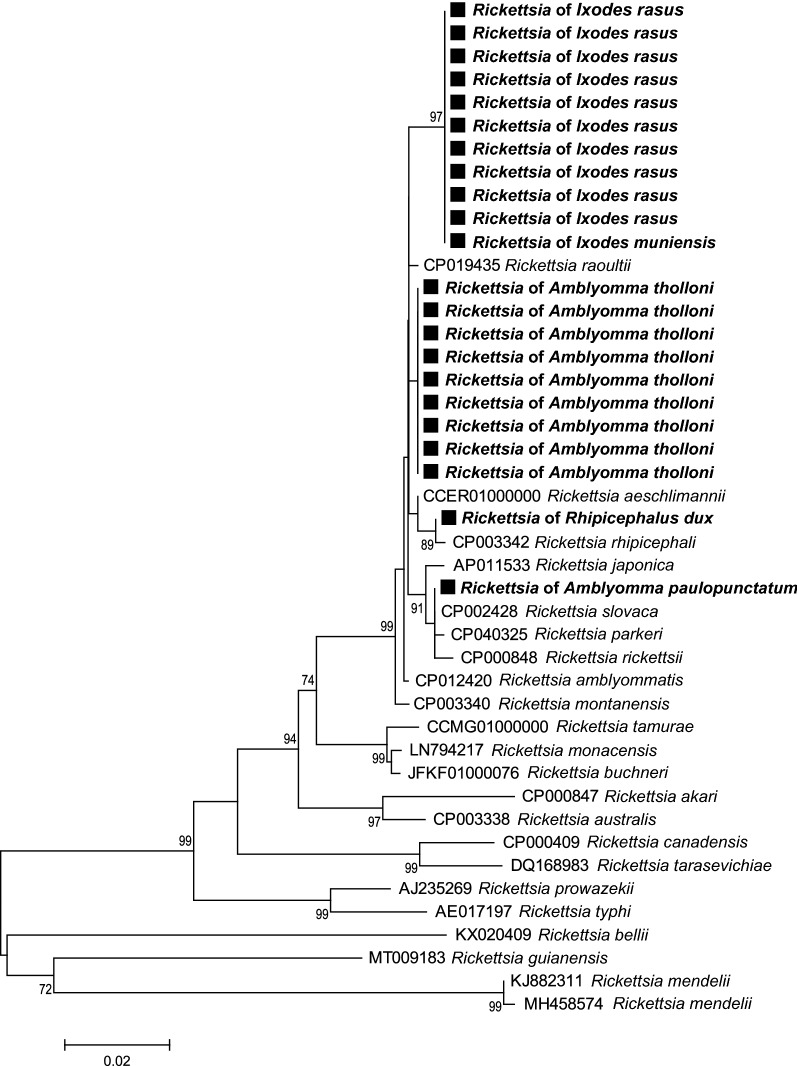
*Rickettsia* phylogenetic tree constructed using maximum-likelihood (ML) estimations based on *gltA* nucleotide sequences (589 unambiguously aligned bp; best-fit approximation for the evolutionary model: T92 + G), including representative *Rickettsia* species (their GenBank accession numbers are indicated on the tree). Branch numbers indicate percentage bootstrap support (1000 replicates). Only bootstrap supports > 70% are shown. The scale bar is in units of substitution/site. Black squares indicate Rickettsia sequences obtained in this study

For piroplasmids, examination of 18S rDNA sequences showed that those of *A. tholloni*, *I. muniensis* and *H. parmata* had moderate levels of pairwise nucleotide identities (88.3%–90.3%). The piroplasmids of these tick species belong to three distinct clades: the first clade includes the six piroplasmids detected in *A. tholloni* (100% pairwise nucleotide identities), which are closely related to *Theileria cervi* and *T. ovis* (98.5% and 97.6% pairwise nucleotide identities, respectively); the second clade includes the piroplasmid detected in *I. muniensis*, which is closely related to *Babesia capreoli* (95% pairwise nucleotide identity); the third clade includes the piroplasmid detected in *H. parmata*, which is closely related to *B. occultans* and *B. orientalis ovis* (94.7% and 95.4% pairwise nucleotide identities, respectively) (Fig. 5). For *Borrelia*, examination of *flaB* gene sequences found in *H. parmata* showed that all cluster together and are closely related to a relapsing fever *Borrelia*, *B. theileri* (98.7% pairwise nucleotide identity) (Fig. 6). For *Cryptoplasma* and *Ehrlichia* (they are sister genera belonging to the Anaplasmataceae family), examination of 16S rDNA sequences showed that the *Cryptoplasma* identified in *H. parmata* and *A. tholloni* (99.2% pairwise nucleotide identity) cluster together with *Cryptoplasma californiense* (98.9% and 99.1% pairwise nucleotide identities, respectively), while the *Ehrlichia* from *H. punctaleachi* are closely related to *E. shimanensis* (98.9% pairwise nucleotide identity) (Fig. 7). For *Rickettsia*, we did not obtain clean *gltA* sequences from *H. parmata*, which did not allow us to include them in the phylogenetic analysis. Examination of *gltA* gene from sequences obtained from other tick species showed that all the *Rickettsia* found in this study belong to the Spotted Fever group (98.7%–99.3% pairwise nucleotide identities): the *Rickettsia* identified in *A. paulopunctatum* is closely related to *R. slovaca* and *R. parkeri* (99.9% and 99.2% pairwise nucleotide identities, respectively), the *Rickettsia* from *Rh. dux* to *R. rhipicephali* (99.2% pairwise nucleotide identity), the *Rickettsia* from *Ixodes muniensis*, *Ixodes rasus* s.l. and *A. tholloni* to *R. raoulti* (99.1% pairwise nucleotide identity), although all are slightly different from these known species (Fig. 8).

## Discussion

In this survey, 470 ticks were collected from the natural forest of a wild chimpanzee community living in 25 km^2^ in western Uganda. We identified seven tick species from four genera. The most common genus was *Haemaphysalis*, and the most frequently collected species were *H. parmata* and *A. tholloni*. The latter species was the only one found inside the fresh nest of a chimpanzee. We found more ticks during the dry than rainy season. The number of ticks collected was influenced by the temperature of the day and the type of vegetation, but not by the relative humidity of the day. Two genera of piroplasmids and four of bacterial pathogens were identified from the adult and nymph ticks using molecular biology. Overall, *Rickettsia* sp. were the most prevalent pathogens in the ticks collected in this study. The individual of *A. tholloni* collected in the nest of a chimpanzee was infected by *Rickettsia* sp.*,* a pathogen also found in ticks collected from the vegetation ground of nesting sites together with *Cryptoplasma* sp*. Theileria* sp. were the third most prevalent pathogens identified in the ticks collected in this study and only found in *A. tholloni* ticks. Ticks collected from the vegetation sites were positive for all pathogens.

Each of the seven species found were hard ticks with a three-host life cycle and can feed on a wide variety of hosts present in the Sebitoli habitat (listed in Additional file 3: Table S2). These tick species feed on large mammals including mostly ruminants, elephants, swines and, to a lesser extent, canids, felids and viverrids. Although some tick species could be more or less specialized to certain animal hosts, like *A. tholloni* with African elephants, other are global generalists and can bite Hominidae, at least occasionally (Additional file 4: Table S2). Two species (*H. parmata* and *I. rasus* s.l.) are known to have non-human primates as hosts, and one species (*I. muniensis*) has Hominidae among its hosts. Five species (*A. paulopunctatum*, *A. tholloni*, *H. parmata*, *I. muniensis* and *I. rasus* s.l.) are known to at least occasionally bite humans and thus might feed on wild chimpanzees. The discovery of *A. tholloni* inside a chimpanzee nest led us to hypothesize that it was either detached by the chimpanzee when self-grooming or was targeting to feed on it as the chimpanzee left the nest which had been constructed the evening before. Usually, no other animal is present in the fresh night nest at the same time as the chimpanzee; thus, the most likely host was the chimpanzee. In addition, the sequence of *A. tholloni* is close to the sequence of an *Amblyomma* sp., which was discovered in the nostrils of primatologists in the Kibale National Park and also appeared to infect the nostrils of chimpanzees living there [30, 31]. As *A. tholloni* adults have specialized in infecting elephants, particularly their trunks [68], and some species have favorite location to bite [69, 70], it is therefore possible that this species or a related species could infect the nostrils of other sympatric animals.

We found that the tick abundances did vary between seasons as expected [71]. The abundance of ticks increased with temperature. This could be attributed to the fact that higher temperatures affect tick activity [46, 72]. Contrary to our expectation, the relative humidity of the day did not significantly affect the activity of ticks. It could be that differences in humidity are not perceptible as the relative humidity is always quite high in Sebitoli as a high-altitude moist forest. As found in other studies [72, 73], the type of vegetation can influence the quantity of tick species that can be collected. We found more ticks in the trail system than in the herbaceous vegetation and secondary forest and finally less in the primary forest. The higher number of ticks found on trails could be due to the high passage of potential hosts on clear paths or to the easier collection because of shorter vegetation. So, humans, by cutting the forest to clear a path, could have an impact on the distribution of ticks.

Because ticks can have different feeding strategies, using multiple techniques wih different biases allowed us to collect a wider variety of tick species and life stages [74, 75]. For example, flagging has been shown to favor larvae and nymphs while visual search favors adult ticks of *Amblyomma* sp. [75]. This could partially explain the high number of larvae and nymphs we collected in this study. Using human subjects favored ticks that feed on humans, while flagging targeted questing ticks with a broader spectrum of hosts [74]. In that regard, *A. paulopuctatum* was collected only from the human members of the research project. It is interesting to note that the nymphal stage of this species was initially assumed to be the tick found in the nostrils of researchers visiting Ugandan forests [32]. Looking inside chimpanzee nests could have allowed for the collection of soft ticks, as they are known to specialize in sheltered microhabitats such as animal nests and burrows [76]. However, chimpanzee nests are temporary structures for one night, unlike others nests where animals may live for several weeks or months. Either the soft ticks could not target the chimpanzee nests or the chimpanzees themselves. This method was very complicated and difficult to implement because it required a fresh nest that could be climbed into, so the number of nests investigated was low. However, it is safe to assume that the tick we found was from or aimed at a chimpanzee, which was valuable information. In addition, Sebitoli chimpanzees have been shown to select certain nesting tree species that have repellent properties against the mosquito *Anopheles gambiae* [77]. This repellent property, although it needs to be tested and confirmed against ticks, could explain the low number of ticks we collected from the nests.

Further examination of microbial communities revealed the presence of six genera of tick-borne pathogens in the seven tick species that were collected in the present study. *Rickettsia* sp. were the most common tick-borne bacteria we detected with 30.91% of ticks infected with it. Some were closely related to virulent pathogens like the *Rickettsia* of *A. paulopunctatum*, which were closely related to *R. parkeri*, the etiological agent of a human rickettsiosis with skin lesions and lymphadenitis. Others were related to *Rickettsia* of unknown pathogenicity as the *R. rhipicephali* of the *Rh. dux* tick. In the Keita and colleagues study [78], of 598 chimpanzee fecal samples, 6.5% were positive for *Rickettsia*, sp. including 2.3% for *R. felis* which was assumed by the authors to have been transmitted by mosquitoes. *Rickettsia* DNA, similar to *R. africae*, an agent of African tick bite fever, was also detected in the blood of primates such as the vervet monkey (*Chlorocebus pygerythrus*) and the yellow baboon (*Papio cynocephalus*) [79]. However, the current view in rickettsiology has a strong anthropocentric bias and tends to describe all novel *Rickettsia* species as pathogenic forms [80, 81]. However, most of the novel *Rickettsia* species or strains discovered in recent years were found exclusively in arthropods and never in vertebrates for which they are not pathogenic [82, 83]. In ticks, as for many other arthropods, some *Rickettsia* sp. are non-infectious agents but maternally inherited endosymbionts with poorly known effects on tick biology as for *R. buchneri* in the black-legged tick *I. scapularis* [84] and *R. vini* in the tree-hole tick *I. arboricola* [85]. In addition, none of these *Rickettsia* sp. were identical to *Candidatus* Rickettsia davousti previously detected in an *Amblyomma* sp. tick collected from the nostril of a national park visitor in Gabon [86]. The prevalence of *Cryptoplasma* sp. was 6.81% in all the ticks with 15.25% in *A. tholloni* and 2.21% in *H. parmata*. *Cryptoplasma* is an enigmatic *Anaplasma*-like pathogen (Anaplasmataceae) that was sporadically identified [87]. Its medical and veterinary importance remains unknown but rodents are potential reservoir hosts [87]. Only few Anaplasmataceae have been previously detected in primates [as in the vervet monkey and baboons (*Papio sp.*)] [79, 88], but *Cryptoplasma* sp. has never been detected in primates or in Africa. The prevalence of piroplasmids was 3.64% in all the tick species with 10.17% in the species of *A. tholloni* only. The prevalence was much lower than in Kenya with *A.tholloni* (51%) and *Rh. humeralis* (27%) but higher than in other species of *Rhipicephalus* (2.7–6%) [89]. Some piroplasmids are responsible for piroplasmosis, a multisystem disease in animals and occasionally humans. *Babesia* infection was detected in the blood of few primates: the indri (*Indri indri*), diademed sifaka (*Propithecus diadema*) [90] and yellow baboon [79]. Similarly, the *Babesia* detected in *I. muniensis* and *H. parmata* are closely related (but different) to species primarily infecting ruminants, *Babesia capreoli* and *B. occultans*/*B. orientalis*, respectively. The *Theileria* sp. found in *A. tholloni* could be specific to ruminants: its phylogenetic proximity to *T. cervi* and *T. ovis*, two species infecting different ruminant species worldwide, suggests that it may have a similar host spectrum. The prevalence of the analyzed samples was 1.82% for *Borrelia* sp., a pathogen related to relapsing fever, detected in *H. parmata*, and closely related to a species infecting bovine *B. theileri*. Previous *Borrelia* infection was only detected once in the blood of the indri primate [90]. The *Ehrlichia* sp. of *H. punctaleachi* was closely related to *E. shimanensis*. *Ehrlichia* sp. DNA was detected in the samples from multiple primates species: the marmosets (*Callithrix* spp.) [91], black lemur (*Eulemur macaco flavifrons*) [92], ring-tailed lemur (*Lemur catta*) [93], langurs (*Semnopithecus* sp.) [94] and black-and-white ruffed lemur (*Varecia variegata*) [92, 93]. The infection rates in ticks for *Ehrlichia* sp. were lower (0.45%) than what was found in ticks of other sites (16.4–18%) [89].

The literature reports a wide variation in pathogen prevalence between animal populations, which may be related to individual host susceptibility, tick preferences and the pathogen species involved. To our knowledge, the only report of tick-borne infection in chimpanzees has been a *Babesia divergens* infection in two splenectomized individuals in Liberia [95]. In Kibale, ticks can be found in the nostrils of both young chimpanzees and researchers [31, 32], highlighting the potential risk of cross-contamination between humans and chimpanzees. In the Sebitoli area, chimpanzees regularly visit surrounding gardens to feed on crops [96], a proximity that could favor the cross-transmission of pathogens as has been shown for malaria [97] and *Oesophagostomum* infection [98].

## Conclusion

We found high number of ticks infested with a high diversity of tick-borne pathogens of human and wildlife concern. Daytime temperature and vegetation type play a role in the probability of encountering ticks and therefore the potentially pathogenic microorganisms they carry. This study was the first to our knowledge to estimate the potential risk of tick-borne pathogen transmission to our closest relative, the threatened chimpanzee, in its natural environment using non-invasive methods. For the first time and thanks to collection in their nests, we found that they are likely hosts for *A. tholloni*, which exposes them to *Rickettsia* sp. pathogens. We also showed that they are at potential risk of encountering *I. rasus* s.l., *A. tholloni* and *H. parmata*, which can be infected with *Rickettsia* sp. and *Cryptoplasma* sp. on the ground around the nest. Further investigations on chimpanzee exposure could collect ticks at other times of the day and on the ground, such as on vegetation after a resting or grooming session. Also, the vegetation used for nesting could be studied to determine whether certain tree species might have repellent properties or favor the unhooking of ticks at night when chimpanzees did not have the opportunity for mutual grooming with conspecifics for up to 12 h. This study further revealed a substantial diversity of tick-borne pathogens, including piroplasmids and bacteria, in Uganda. This underlined the need to better document the diversity of ticks and tick-borne pathogens in natural habitats of an endangered species such as the chimpanzees. In Uganda, the effect of these tick-borne pathogens on animal health remains to be elucidated.

## Supplementary Information

**Additional file 1: Table S1.** List of specific primers and PCR conditions used to detect tick-borne pathogens. Semi-nested PCR amplifications were performed as follows: the first PCR run with the external primers was performed in a 10-μl volume containing 10–50 ng of genomic DNA, 3 mM of each dNTP (Thermo Scientific), 8 mM MgCl2 (Roche Diagnostics), 3 μM of each primer, 1 μl 10× PCR buffer (Roche Diagnostics) and 0.5 U Taq DNA polymerase (Roche Diagnostics). A 1-μl aliquot of the PCR product from the first reaction was used as a template for the second round of amplification. The second PCR was performed in a total volume of 25 μl and contained 8 mM of each dNTP (Thermo Scientific), 10 mM of MgCl2 (Thermo Scientific), 7.5 μM of each of the internal primers, 2.5 μl of 10× PCR buffer (Thermo Scientific) and 1.25 U Taq DNA polymerase (Thermo Scientific). All PCR amplifications were performed as follows: initial denaturation at 93 °C for 3 min, 35 cycles of denaturation (93 °C, 30 s), annealing (Tm = 52–56 °C depending on primers, 30 s), extension (72 °C, 1 min) and a final extension at 72 °C for 5 min.

**Additional file 2: Figure S1. **Dorsal and ventral pictures of remarkable tick individuals collected at Sebitoli, Kibale National Park, Uganda.

**Additional file 3: Table S2.** Information on species of ticks collected and their hosts according to literature (Guglielmone et al. 2014; Hoogstraal and Theiler, 1959; Ntiamoa-Baidu et al. 2004). A: adult stage, N: nymphal stage, L: larval stage. In bold, family of hosts found in Sebitoli (incertitude with birds and snakes).

**Additional file 4: Table S2.** Information on species of ticks collected and their hosts according to literature (Guglielmone et al. 2014; Hoogstraal and Theiler, 1959; Ntiamoa-Baidu et al. 2004). A: adult stage, N: nymphal stage, L: larval stage. In bold, family of hosts found in Sebitoli (incertitude with birds and snakes).

## Data Availability

All data generated or analyzed during this study are included in this published article and its supplementary information files.
