# Supplementary material for: Survey of ticks and tick-borne pathogens in wild chimpanzee habitat in Western Uganda
Source: Parasit Vectors. 2023 Jan 22;16:22. doi: 10.1186/s13071-022-05632-w (PMC9869571; doi:10.1186/s13071-022-05632-w)
Supplement: Supplementary file 1 — Additional file 1: Table S1. List of specific primers and PCR conditions used to detect tick-borne pathogens. Semi-nested PCR amplifications were performed as follows: the first PCR run with the external primers was performed in a 10-μl volume containing 10–50 ng of genomic DNA, 3 mM of each dNTP (Thermo Scientific), 8 mM MgCl2 (Roche Diagnostics), 3 μM of each primer, 1 μl 10× PCR buffer (Roche Diagnostics) and 0.5 U Taq DNA polymerase (Roche Diagnostics). A 1-μl aliquot of the PCR product from the first reaction was used as a template for the second round of amplification. The second PCR was performed in a total volume of 25 μl and contained 8 mM of each dNTP (Thermo Scientific), 10 mM of MgCl2 (Thermo Scientific), 7.5 μM of each of the internal primers, 2.5 μl of 10× PCR buffer (Thermo Scientific) and 1.25 U Taq DNA polymerase (Thermo Scientific). All PCR amplifications were performed as follows: initial denaturation at 93 °C for 3 min, 35 cycles of denaturation (93 °C, 30 s), annealing (Tm = 52–56 °C depending on primers, 30 s), extension (72 °C, 1 min) and a final extension at 72 °C for 5 min. [file 13071_2022_5632_MOESM1_ESM.docx]

**Additional file 1: Table S1.** List of specific primers and PCR conditions used to detect tick-borne pathogens. *Semi-nested PCR amplifications were performed as follows: the first PCR run with the external primers was performed in a 10 μLvolume containing 10–50 ng of genomic DNA, 3 mM of each dNTP (Thermo Scientific), 8 mM of MgCl2 (Roche Diagnostics), 3 μM of each primer, 1 μL of 10× PCR buffer (Roche Diagnostics), and 0.5 U of Taq DNA polymerase (Roche Diagnostics). A 1-μL aliquot of the PCR product from the first reaction was used as a template for the second round of amplification. The second PCR was performed in a total volume of 25 μL and contained 8 mM of each dNTP (Thermo Scientific), 10 mM of MgCl2 (Thermo Scientific), 7.5 μM of each of the internal primers, 2.5 μL of 10× PCR buffer (Thermo Scientific), and 1.25 U of Taq DNA polymerase (Thermo Scientific). All PCR amplifications were performed as follows: Initial denaturation at 93°C for 3 min, 35 cycles of denaturation (93°C, 30 s), annealing (Tm=52-56°C depending on primers, 30 s), extension (72°C, 1 min), and a final extension at 72°C for 5 min.*

| **Tick-borne pathogens** | **Gene** | **Product** | **Primers (5'-3')** | | **Tm** | **Fragment size** | **Reference for primers** |
| --- | --- | --- | --- | --- | --- | --- | --- |
| Piroplasmids | 18S rRNA | Small ribosomal subunit (SSU rRNA) | Bab310F1 | GCCTACCGRGGCARCAACG | 54°C | 1st round PCR: Bab310F1 + Bab993R2 : 691-734bp  2nd round PCR (semi-nested PCR): Bab579F2 + Bab993R2 : 437-479bp | This study |
|  |  |  | Bab579F2 | GCGTATATTAAASTTGTTGCAG |  |  |  |
|  |  |  | Bab993R2 | GATTTCTCTCAAGSTSCTGAAG |  |  |  |
| *Rickettsia* | *glta* | Citrate synthetase | RickF1 | GTTCTCTTTCKGCATTTTATCC | 56°C | 1st round PCR: RickF1 + RickR1: 672bp  2nd round PCR(semi-nested PCR): RickF2 + RickR1: 645bp | (Duron et al., 2017) |
|  |  |  | RickF2 | GTTCTCTTTCKGCATTTTATCC |  |  |  |
|  |  |  | RickR1 | CATCTTTAAGAGCGATAGCTTCAAG |  |  |  |
| *Borrelia* | *flaB* | Flagellin | FlaB_F2 | CTGATGATGCTGCTGGWATGGG | 52°C | 1st round PCR: FlaB_F2 + FlaB_R1 : 910bp  2nd round PCR (semi-nested PCR): FlaB_F2 + FlaB_R2 : 644bp | This study, adapted from (Binetruy et al., 2020) |
|  |  |  | FlaB_R1 | GCAATCATAGCCATTGCRG |  |  |  |
|  |  |  | FlaB_R2 | CCACCTTGARCTGGAGC |  |  |  |
| *Ehrlichia* and *Cryptoplasma* | 16S rRNA | Small ribosomal subunit (SSU rRNA) | Ehr16S_F1 | TCGCTATTAGATGAGCCTA | 52°C | 1st round PCR: Ehr16S_F1 + Ehr16S_R2 : 1200bp  2nd round PCR (semi-nested PCR, fragment 1): Ehr16S_F1 + Ehr16S_R1 : 796bp  2nd round PCR (semi-nested PCR, fragment 2): Ehr16S_F2 + Ehr16S_R3 : 457bp | This study |
|  |  |  | Ehr16S_F2 | TGACATGAAGGTCGTATCC |  |  |  |
|  |  |  | Ehr16S_R1 | GGTCCAGCCGAACTGACTC |  |  |  |
|  |  |  | Ehr16S_R2 | AGCACACCAGCTTCGAGTTA |  |  |  |
|  |  |  | Ehr16S_R3 | AGTTAAGCCAATTCCCATGG |  |  |  |

Binetruy, F., Garnier, S., Boulanger, N., Talagrand-Reboul, E., Loire, E., Faivre, B., Noël, V., Buysse, M., Duron, O., 2020. A novel *Borrelia* species, intermediate between Lyme disease and relapsing fever groups, in neotropical passerine-associated ticks. Sci. Rep. 10, 1–10. https://doi.org/10.1038/s41598-020-66828-7

Duron, O., Binetruy, F., Noël, V., Cremaschi, J., McCoy, K.D., Arnathau, C., Plantard, O., Goolsby, J., Pérez de León, A.A., Heylen, D.J.A., Van Oosten, A Raoul Gottlieb, Y., Baneth, G., Guglielmone, A.A., Estrada-Peña, A., Opara, M.N., Zenner, L., Vavre, F., Chevillon, C., 2017. Evolutionary changes in symbiont community structure in ticks. Mol. Ecol. 26, 2905–2921. https://doi.org/10.1111/ijlh.12426
