# Supplementary material for: Survey of ticks and tick-borne pathogens in wild chimpanzee habitat in Western Uganda
Source: Parasit Vectors. 2023 Jan 22;16:22. doi: 10.1186/s13071-022-05632-w (PMC9869571; doi:10.1186/s13071-022-05632-w)
Supplement: Supplementary file 2 — Additional file 2: Figure S1. Dorsal and ventral pictures of remarkable tick individuals collected at Sebitoli, Kibale National Park, Uganda. [file 13071_2022_5632_MOESM2_ESM.pdf]

Figure S1. Dorsal and ventral pictures of remarkable tick individuals collected at Sebitoli, Kibale National Park, Uganda.

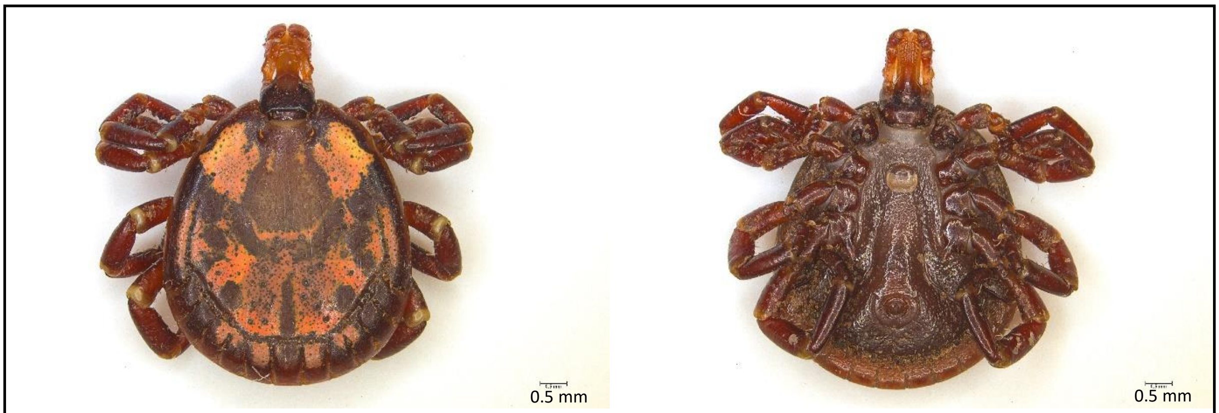

Amblyomma paulopunctatum – Adult male

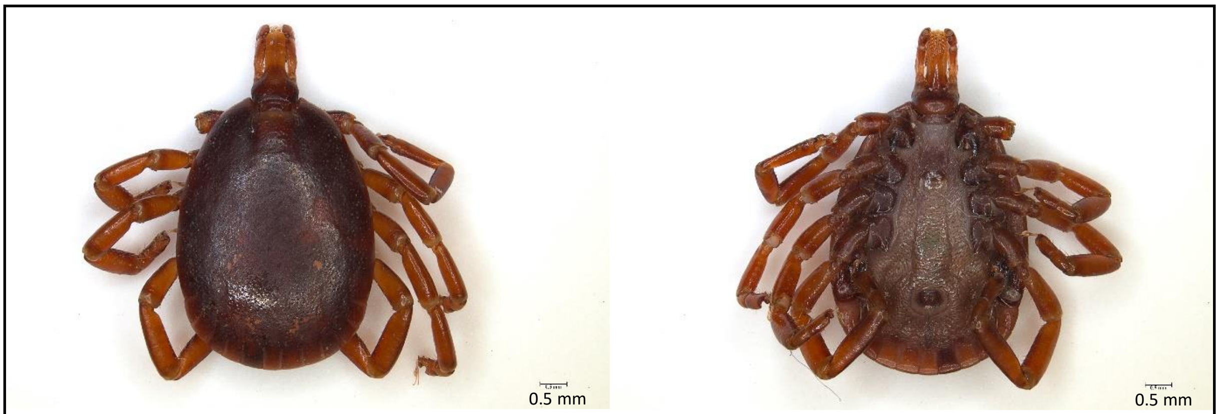

Amblyomma tholloni – Adult male

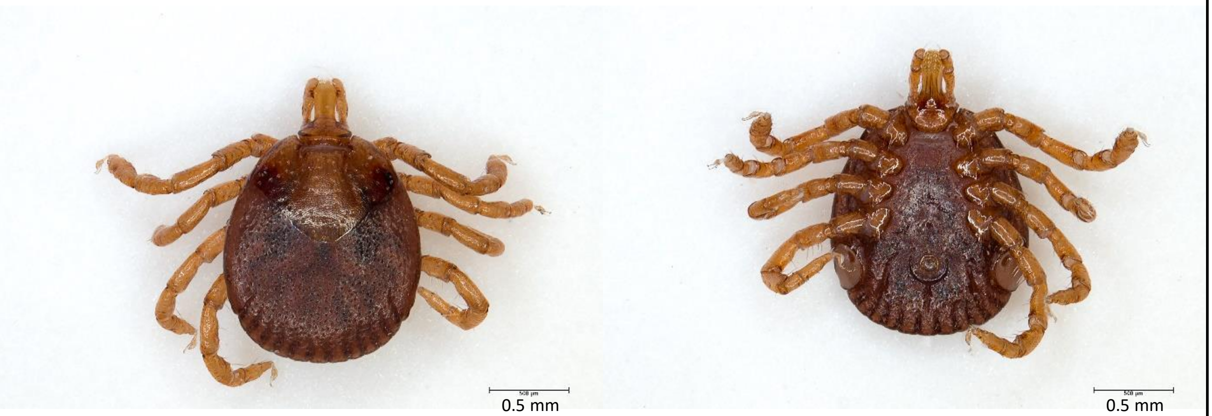

Amblyomma tholloni – Nymph

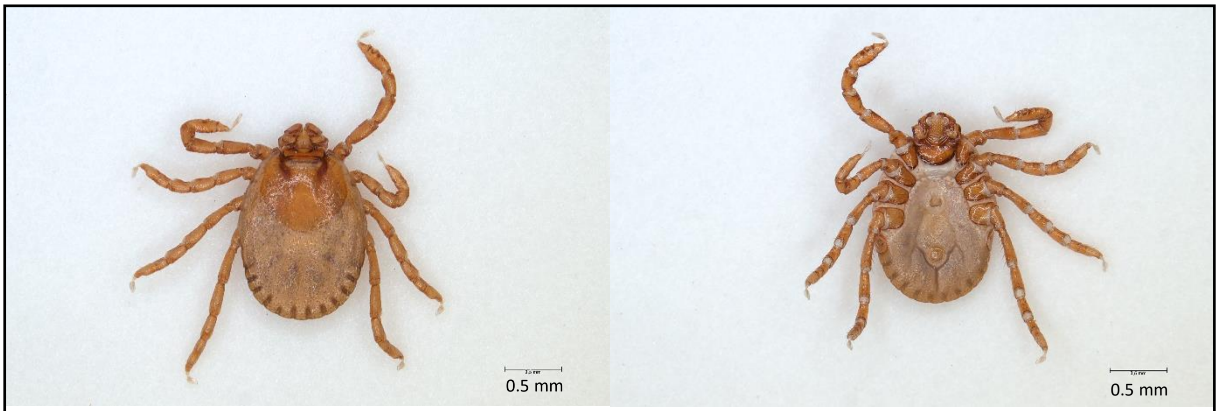

Haemaphysalis parmata – Adult female

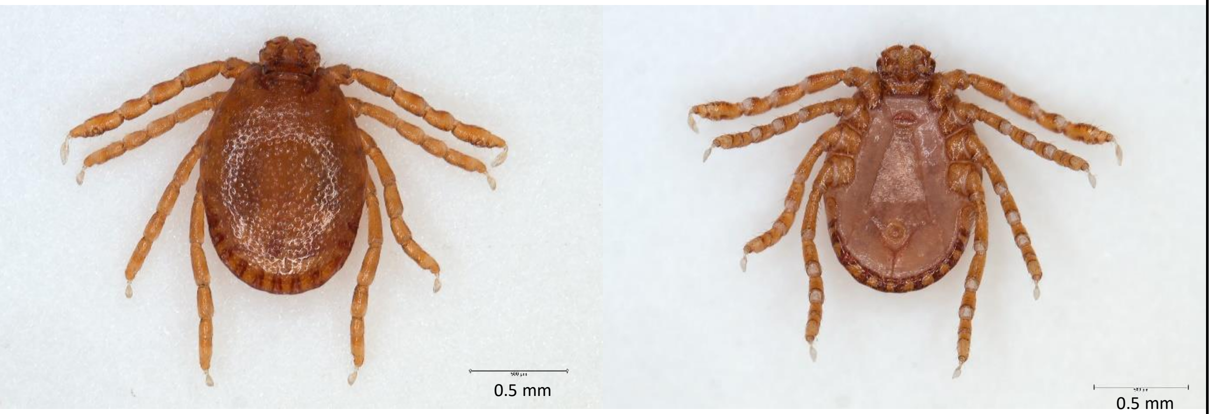

Haemaphysalis parmata – Adult male

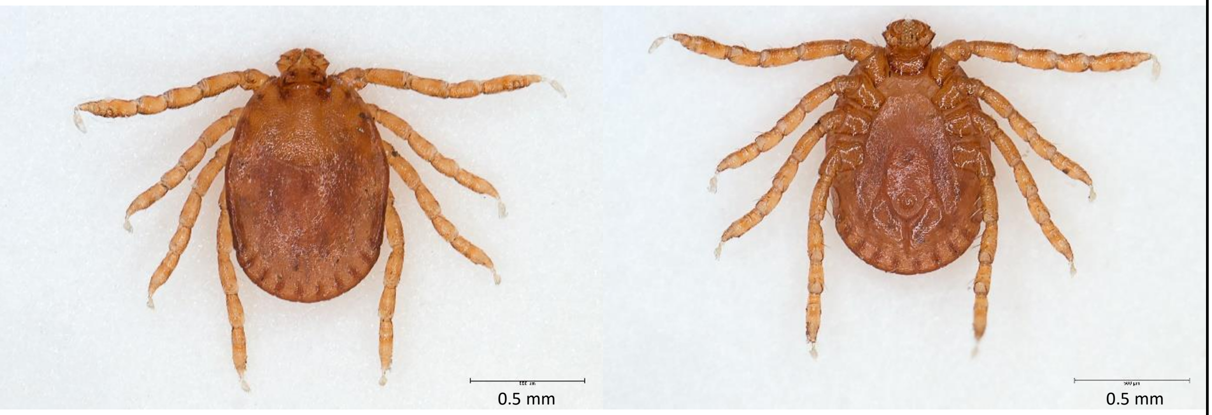

Haemaphysalis parmata – Nymph

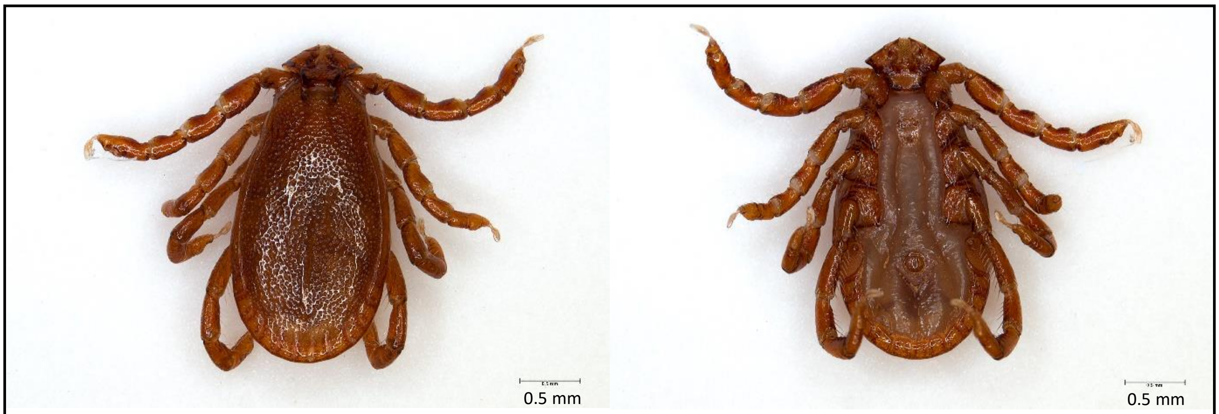

Haemaphysalis punctaleachi – Adult female

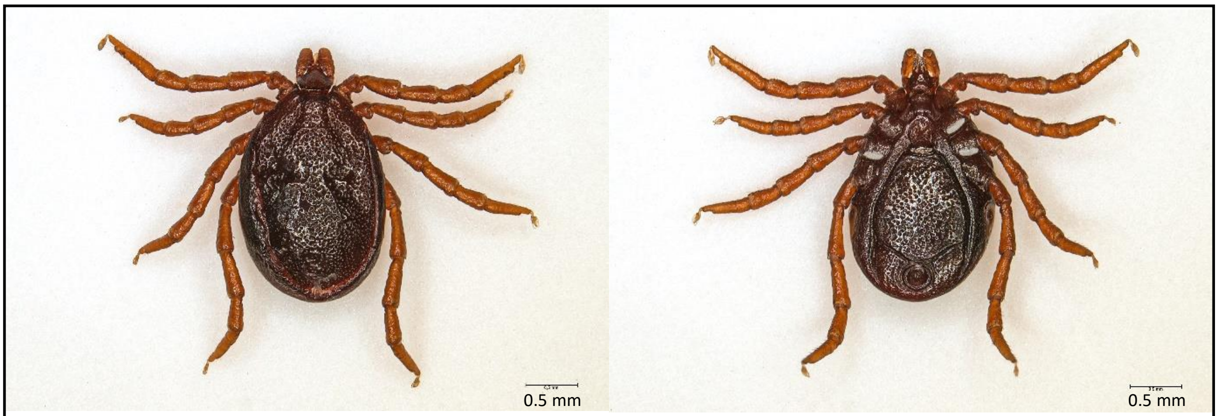

Ixodes muniensis – Adult male

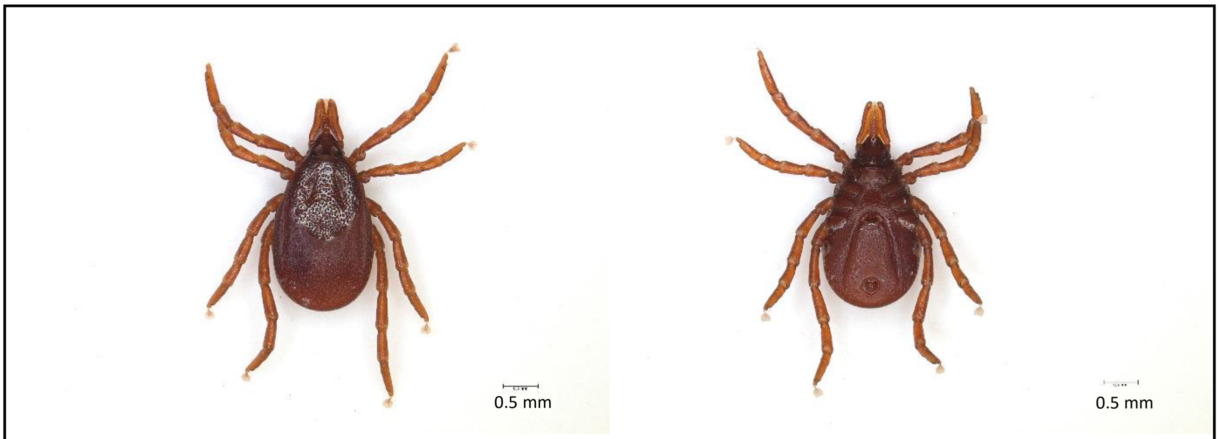

Ixodes rasus/latus – Adult female

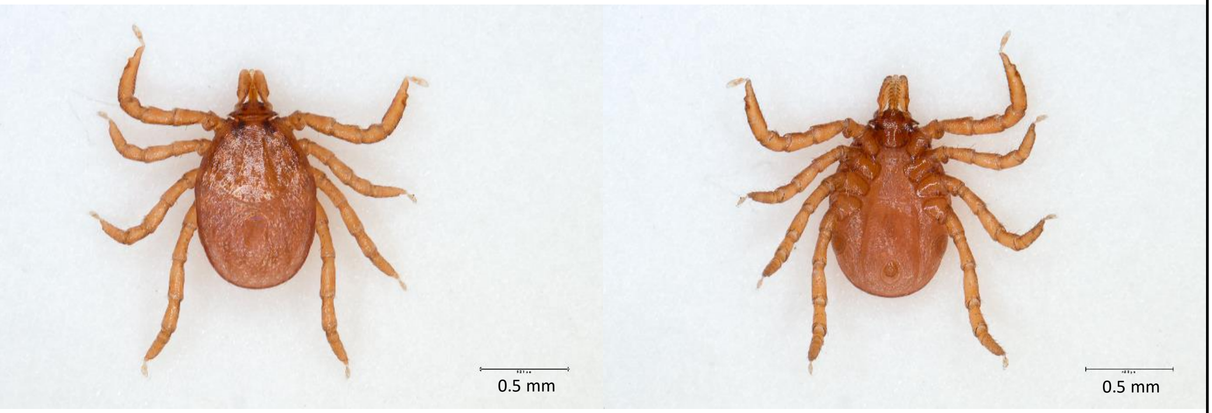

Ixodes rasus/latus – Nymph

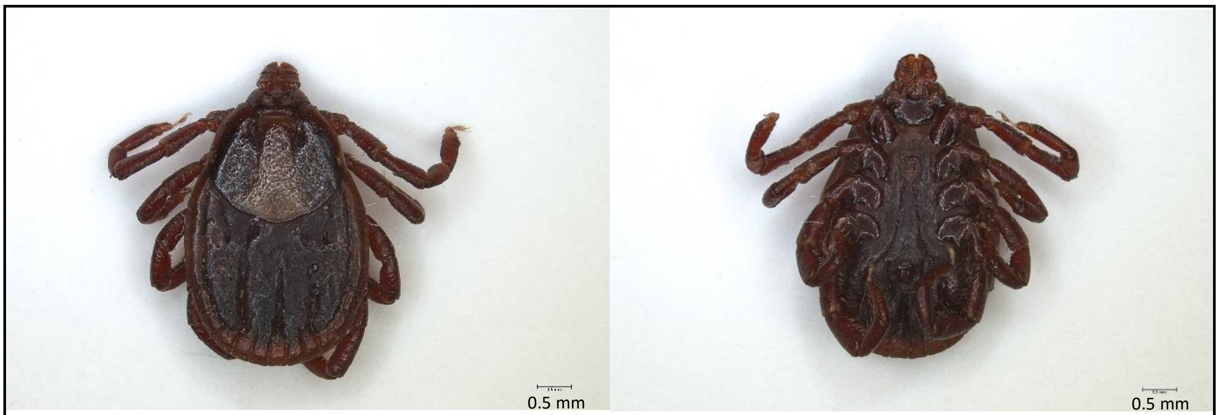

Rhicephalus dux – Adult female

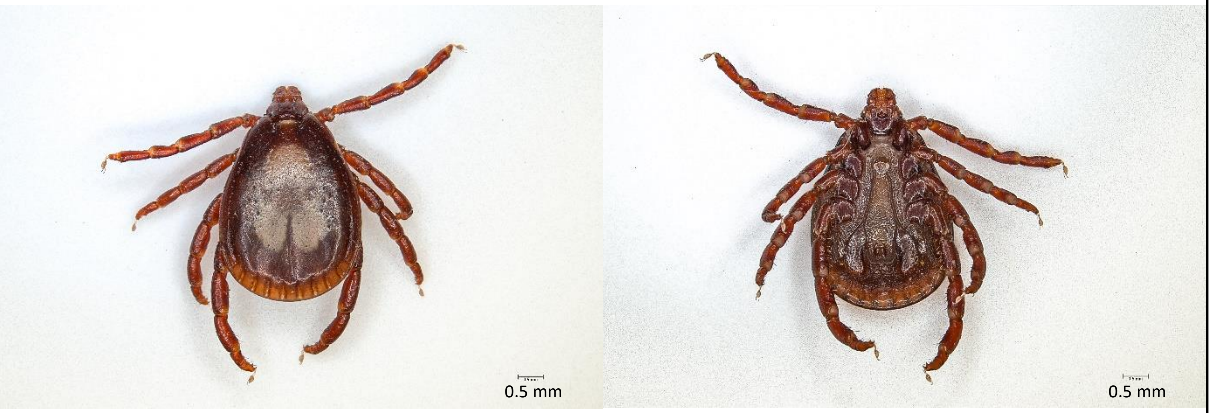

Rhicephalus dux – Adult male
