# Supplementary material for: Survey of ticks and tick-borne pathogens in wild chimpanzee habitat in Western Uganda
Source: Parasit Vectors. 2023 Jan 22;16:22. doi: 10.1186/s13071-022-05632-w (PMC9869571; doi:10.1186/s13071-022-05632-w)
Supplement: Supplementary file 3 — Additional file 3: Table S2. Information on species of ticks collected and their hosts according to literature (Guglielmone et al. 2014; Hoogstraal and Theiler, 1959; Ntiamoa-Baidu et al. 2004). A: adult stage, N: nymphal stage, L: larval stage. In bold, family of hosts found in Sebitoli (incertitude with birds and snakes). [file 13071_2022_5632_MOESM3_ESM.docx]

**Additional file 3: Table S2.** Information on species of ticks collected and their hosts according to literature (Guglielmone et al., 2014; Hoogstraal and Theiler, 1959; Ntiamoa-Baidu et al., 2004). *A: adult stage, N: nymphal stage, L: larval stage. In bold, family of hosts found in Sebitoli (incertitude with birds and snakes).*

| **Species** | **Hosts** |
| --- | --- |
| *Amblyomma paulopunctatum* Neumann, 1899 | **Suidae** (A/N/L); **Bovidae** (A/N); Hippopotamidae (A); Hyaenidae (A); Thryonomyidae (A); **Phasianidae** (N/L) + **Human** |
| *Amblyomma tholloni* Neumann, 1899 | **Bovidae** (A/N/L); Hippopotamidae (A/N/L); **Elephantidae** (A/N/L); **Pittidae** (A/N/L); Testudinidae (A/N/L); **Suidae** (A/N); **Felidae** (A/N); **Canidae** (A); **Herpestidae** (A); Equidae (A); Rhinocerotidae (A); Accipitridae (N); **Musophagidae** (N) + **Human** |
| *Haemaphysalis parmata* Neumann, 1905 | **Bovidae** (A); **Phasianidae** (A/N/L); **Numididae** (A/N/L); Hystricidae (A/N); Elapidae (A); **Herpestidae** (A); **Nesomyidae** (A); Thryonomyidae (A); **Viverridae** (A); **Cercopithecidae** (N); **Estrildidae** (N); Viduidae (L); Atractaspididae (L); Bucorvidae (N/ L) + **Human** |
| *Haemaphysalis punctaleachi* Camicas, Hoogstraal & El Kammah, 1973 | **Viverridae** (A); **Bovidae** (A); Hystricidae (A) |
| *Ixodes muniensis* Arthur & Burrow, 1957 | **Bovidae** (A/N/L); **Canidae** (A/N/L); **Herpestidae** (A/N/L); **Nesomyidae** (A/N/L); Giraffidae (A); **Suidae** (A); **Viverridae** (A); **Felidae** (A/L); Procaviidae (N); **Hominidae** (N); **Muridae** (L); **Sciuridae** (L) + **Human** |
| *Ixodes rasus* senu lato (s.l) Neumann, 1899 | **Bovidae** (A/N/L); **Canidae** (A/N/L); **Herpestidae** (A/N/L); **Cercopithecidae** (A); **Muridae** (N/L); **Sciuridae** (N/L); Numididae (N/L); Procaviidae (N); **Phasianidae** (N); Cuculidae (N); Turdidae (L) + **Human** |
| *Rhipicephalus dux* Dönitz, 1910 | **Bovidae** (A); **Suidae** (A); **Elephantidae** (A) |

Guglielmone, A.A., Apanaskevich, D.A., Estrada-Peña, A., Robbins, R.G., Petney, T.N., Horak, I.G., 2014. The hard ticks of the world: (Acari: Ixodida: Ixodidae), The Hard Ticks of the World: (Acari: Ixodida: Ixodidae). https://doi.org/10.1007/978-94-007-7497-1

Hoogstraal, H., Theiler, G., 1959. Ticks (Ixodoidea, Ixodidae) parasitizing lower primates in Africa, Zanzibar, and Madagascar. J. Parasitol. 45, 217–222.

Ntiamoa-Baidu, Y., Carr-Saunders, C., Matthews, B.E., Preston, P.M., Walker, A.R., 2004. An updated list of the ticks of Ghana and an assessment of the distribution of the ticks of Ghanaian wild mammals in different vegetation zones. Bull. Entomol. Res. 94, 245–260. https://doi.org/10.1079/ber2004302
